# Medical Items, News, and Gossip

**Published:** 1870-12

**Authors:** 


### Medical Items, News, and Gossip.

E. M., a clerk in a public office, came for advice, complaining of excessive flow of urine, with great thirst, voracious appetite, and gradual emaciation. Sp. grav. of urine ranging from 1.030 to 1.040. Various medication, meat diet, etc., were adopted, with never more than temporary relief. On careful inquiry as to the surroundings, it was ascertained that, in the intervals of duty (delivering of letters at the post office window), he was in the habit of stepping back from the opening, and leaning against the tubes of the steam heating apparatus, whereby the back was strongly heated. This habit had been kept up for several months, the weather having been unusually cold. Discontinuance of the practice was enjoined, and perfect recovery ensued, without any further medication or change of diet. The writer, who attended the case, thinks that if he had taken the patient out of the office, and had exhibited some particular medicine, the latter would have had the credit of the cure. There has been no return of the disease, although about seven years have elapsed.——Several of our friends complain of the uncertain action of that usually mild laxative, the citrate of magnesia. It is often inefficient, and often excessively irritant. It varies in its chemical composition, as ordinarily put up in solution, and is both inconvenient and expensive for country use. Ten or twelve years since, our attention was called to this matter by some writer, we think in the *Am. Jour. of Pharmacy*, and we were led to substitute for the citrate of magnesia, the tartrate of soda, which is fully as efficient as the former, even when properly prepared; not liable to decomposition, cheaper, and readily presented in an equally agreeable and palatable solution. It may be extemporaneously given in water, with lemon syrup, or, if desired, a little citric acid may be added. This salt is preferable to the double tartrate of soda and potash in most cases when the latter is employed.——When creasote is indicated in dyspeptic cases, an old and useful addition is Sp. Ammon. Aromat., 15 or 20 drops of the latter to one or two of the former, in an ounce of water. It is better without the syrup usually added.——Trousseau commended as a substitute for cod liver oil, for daily use, four ounces of fresh butter, with three-fourths of a grain of iodide of potassium, three grains of the bromide of potassium, and half a drachm of salt, to be eaten on bread. The medicines, in that butter, would scarcely hurt much, at least in these days, when drachm doses of the bromide are considered "quite the thing." ——The Prussian government has already ordered 200,000 wooden legs.——At Painesville, Ohio, a man lying sick with typhoid fever, was instantly killed by the shock of the recent great explosion of nitro-glycerin, although two miles distant from the point where it occurred. A physician twelve miles distant was stunned by the concussion, his horse brought to a standstill, and his watch stopped in his pocket. The *Scientific American* calls for stringent legislation to prohibit the general use of this fearfully potent and destructive agent.——Houzeau has contrived an apparatus, by aid of which he is able to prepare ozone in any quantity from the air or oxygen. He has not yet given a description of his invention, although publishing the general result of his observation. Considering the intense activity of this great atmospheric purifier, such an invention is attended with much general and practical interest.——At the Children's Hospital of Lausanne, M. Joel treats extensive burns by placing the patient, for fifteen or twenty minutes at a time, in a bath containing a couple of handsfull of sulphate of iron. This is repeated twice a day, and is said to moderate the suppuration, remove the fetid odor, and promote rapid recovery.——The papers say, that the drugging of drinks, whereby the operations of the *chevaliers d'industrie* of our cities are facilitated, is accomplished simply by putting tobacco snuff in the beverage. We are inclined to think that the principal agent in most of these cases is alcohol. ——The *Pacific Journal* advises, for fetid expectoration: ℞ Acid. Carbol., gr. xl; Aq., ℥viij; M.; give a tablespoonful every two or three hours. Or, ℞ Creasoti, gtt. xx; Acid. Sulph. Aromat., ʒ j; Aq., ℥ viij; M.; give in the same way. Or, ℞ Potassæ Permang., gr. viij; Aq., ℥ viij; M.; same directions. The last prescription is reputed to be much the best.——The *Oregon Reporter* states, that turpentine is an antidote for phosphorus. "Let all remember this when a child accidentally helps itself to matches."——Chloride of potassium is recommended by Dr. Sanders as a substitute for the bromide.——Epistaxis is said to be controlled by pressure of a branch of the facial artery, near the ala of the nostril whence the blood is flowing. A slight intermittent movement of the soft parts may be seen or felt by careful observation. So writes a correspondent of the *Gazette des Hopitaux.*——A case of simultaneous intra and extra uterine pregnancy was reported to the Washtenaw (Mich.) Co. Med. Soc. by Prof. Sager. The pathological specimen was forwarded to him by Dr. H. B. Landon, of Bay City. There was a fœtus in the uterine cavity, and its twin in the left Fallopian tube.——A correspondent gives the following as the original VELPEAU'S MIXTURE: ℞ Tinct. Opii, Tinct. Camph. Co., Tinct. Rhei, āā ℥ j; Ess. Menth. Pip., ʒ x; Tinct. Capsici, ʒ vj; M. Dr. Squibb's notorious compound (prescribed in New York city as Tinct. Opii Comp.) is an imitation of this, and from the similarity of the name given it to that by which paregoric is often known, is certainly sufficiently dangerous.——The New York State Inebriate Asylum, at Binghampton, is now under the charge of Dr. Daniel E. Dodge, who may be addressed for information.——The process of skin grafting over extensively denuded and granulating surfaces, suggested as a new method in the Parisian, is essentially a revival of an old practice.——A paper on the Effects of Increased Atmospheric Pressure, by Dr. E. A. Clark, of St. Louis, was accidentally mislaid, but will receive further notice in an ensuing No.

---

***New Polyscope.***

M. Trouve's new polyscope serves for a laryngoscope, ophthalmoscope, otoscope, and urethroscope, and represents, when closed, a case seven inches long by one inch and a quarter in diameter. The two parts composing it carry each a lens at their opposite extremities—the one two and a half inches, and the other three and a half. In the lids which close the case, two mirrors are placed, the one plane, the other concave, both being pierced in the centre. The case contains—1. Two larynx mirrors with handle. 2. Three ear speculums. 3. A photophor, or candlestick, with three branches, terminating on the side of the light by a vent, which at the same time does for a reflector; the photophor can ascend to the height of fifteen and three-quarter inches.

